# Novel Non-Evaporable Getter Materials and Their Possible Use in Fusion Application for Tritium Recovery

**DOI:** 10.3390/molecules25235675

**Published:** 2020-12-01

**Authors:** Alessia Santucci, Luca Farina, Silvano Tosti, Antonio Frattolillo

**Affiliations:** ENEA, Fusion and Technology for Nuclear Safety and Security Department, Via E. Fermi 45, 00044 Frascati, Italy; luca.farina@enea.it (L.F.); silvano.tosti@enea.it (S.T.); antonio.frattolillo@enea.it (A.F.)

**Keywords:** non-evaporable getter, tritium recovery, hydrogen adsorption

## Abstract

Non-evaporable getters (NEGs) are metallic compounds of the IV group, particularly titanium and/or zirconium-based alloys and are usually used as pumps in vacuum technologies since they are able to sorb, by chemical reactions, most of the active gas molecules, with particular efficacy towards hydrogen isotopes. This work suggests an alternative application of these materials to fusion nuclear reactors, where there is the need to recover small amount of tritium from the large helium flow rate composing the primary coolant loop. Starting from the tritium mass balance inside the primary coolant loop, the amount of coolant to be routed inside the coolant purification system (CPS) is identified. Then a feasibility study, based on the bulk getter theory, is presented by considering three different commercial alloys, named ST707, ST101 and ZAO. The results provide the mass, the area and the regeneration parameters of the three different alloys necessary to fulfill the requirements of the CPS unit. By comparing the features of the three alloys, the ZAO material appears the most promising for the proposed application because it requires the lower amount of material and a lower number of regeneration cycles.

## 1. Introduction

Getter materials are able to chemically adsorb gas molecules on their surface, therefore are widely used in the Ultra and Extreme High Vacuum (UHV and XHV) applications. A fundamental aspect for the getter operation is the activation process necessary to create a clean surface on which the active gases (typically H_2_, H_2_O, CO, CO_2_, N_2_, O_2_) can react [1]. According to the procedure used to realize the active surface, getter materials can be classified in two categories: evaporable and non-evaporable getters [2]. Common getter materials used in the evaporated mode are alloys based on barium and titanium which are deposited via evaporation process to form a thin film getter. In case of non-evaporable getters (NEGs) the activation process consists of a heat treatment under dynamic vacuum or under inert (noble gases) atmosphere, in order to remove the passivation layer (mainly oxides and carbides) covering its surface by promoting their bulk diffusion. Materials used as NEGs are metal alloys of the IV group (particularly titanium and zirconium) which exhibit the following characteristics: (i) their oxides easily diffuse at high temperature, ensuring a clean and highly reactive surface and (ii) their chemical activity vs. the active gases usually present in vacuum devices is high [3,4,5]. Non-evaporable getter alloys are able to sorb, by chemical reactions, most of the active gas molecules, with particular efficacy towards hydrogen isotopes. On the contrary, rare gases are not sorbed at all, since they simply do not react. Once the getter is activated, sorption of active gas molecule occurs in a few steps: (i) the first step consists in the dissociation of gas molecules at the getter surface; (ii) dissociation is then followed by sticking of the resulting atomic species onto the getter surface (with a probability that depends on the getter material and temperature and on the nature of the particular gas), this second step always occurs for any type of active gas, though with different binding energies; (iii) the third step consists of the diffusion of adsorbed atoms from underneath the surface to the still empty part of the bulk alloy; it may or may not occur, depending on the diffusivity of each specific gas inside the metal and on the getter temperature. If surface adsorption (steps a and b) is faster than bulk diffusion, the sorption rate is limited by this latter phenomenon; similarly, when diffusion is faster, the sorption rate is limited by the surface kinetics. These two different situations are usually referred to as “diffusion-limited” and “surface-limited” sorption. Actually, when the getter is exposed to a mixture of active and noble gases, a further step should be considered, which is preliminary to the three steps described above, consisting in the diffusion of active molecules across a thin gas layer surrounding the getter surface. This process is driven by the concentration gradient of the active molecular species between the gas bulk (outside of the layer) and the getter surface [6]. The pumping speed, for each gas species, depends of course on which of all the above steps controls the rate.

As reported by Maccallini et al. [2], NEGs are widely used as pumps in industry, research and development (R&D) labs, research centers and large physics projects like accelerators, synchrotrons or fusion reactors [7,8]. Usually NEGs are preferred to others UHV and XHV systems (like turbo molecular, cryogenic or sputter-ion pumps) in those cases in which it is necessary to avoid vibration and generation of electromagnetic disturbances and when the space requirement is an issue. In addition, NEG pumps remove very efficiently hydrogen, that is one of the residual gas in UHV, they do not require any power supply during gas adsorption and they are oil-free. Due to these characteristics, novel getter materials have been recently developed to address the peculiar needs of fusion applications. An example is given by the ZAO^®^ alloy which is under investigation as possible candidate pumping material in the neutral beam injector of the EU-DEMO (European DEMOnstration nuclear fusion power plant) [9,10]. The promising results obtained in the initial characterization campaign has fostered the possibility of using the NEGs technology and in particular ZAO alloy, for the removal of hydrogen isotopes impurities from the helium primary coolant loop [11].

This work identifies another possible application of NEGs inside the fusion technologies. As illustrated in the following, some tritium can permeate into the primary coolant of the EU-DEMO reactor and, from there, it has to be efficiently removed. The conventional process used to remove tritium impurities from the primary coolant loop relies on an oxidation step to form tritiated water followed by the capture and the subsequent processing of such water [12,13]. Without going into details, it is evident that such conventional process requires long processing time and foresees, as intermediate product, the formation of tritiated water whose radiological hazard is much higher than molecular tritium.

Using bulk getter theory, a feasibility study is carried out on three commercial NEG alloys. The results show that an appropriate dimensioning of the coolant purification system relying on the use of NEGs technology is able to ensure a tritium concentration inside the coolant loop within the allowable value.

## 2. The Problem of Tritium Impurities Inside the Helium Primary Coolant of DEMO Fusion Reactor

The EU-DEMO is the nearest-term fusion reactor designed to produce electricity and to operate with a closed fuel-cycle. It represents a machine between ITER, the world’s largest fusion experiment currently under construction in Cadarache [14] and the first-of-a-kind fusion power plant. The reference reaction for this kind of reactors is the one among two hydrogen isotopes, deuterium and tritium, as shown in Equation (1).

(1) D12+T13→H24e(3.52 MeV)+ n01(14.06 MeV).

A cooling medium (helium or water) is used to remove the fusion heat and convert it into electricity. The presence of tritium in Equation (1) involves two problems: one related with its scarcity and one with its radioactivity—tritium is a low beta emitter with half-life of 12.3 years. For such reasons, DEMO must have a closed fuel-cycle to recover, process and reuse either the unburnt tritium or the tritium opportunely produced inside the breeding blanket. Particularly the breeding blanket is a region surrounding the tokamak composed of a Li-based material (there are several options under investigation for the most appropriate blanket configuration, see Reference [15] where the neutrons interact with lithium for producing tritium, see Equation (2).

(2) L36i+n01→H24e(2.05 MeV)+ T13(2.75 MeV).

**Figure 1:**
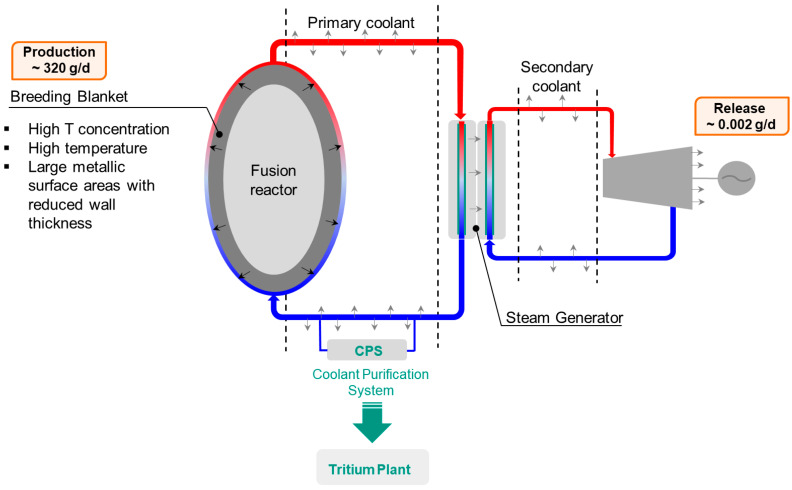
Simplified scheme of the coolant loop and Coolant Purification System (CPS) used for tritium mass balance.

An effective breeding blanket, coupled with an efficient fuel cycle, will allow the DEMO reactor to achieve the ambitious goal of self-sufficiency, meaning that all the reacted tritium is reproduced on site. An accurate tritium control and management is fundamental also for safety aspects; it is worth to mention that the allowable tritium release into environment is currently set at 1 gr per year while the tritium produced inside the breeding blanket is about 320 gr per day—the indicated tritium production rate is referred to a DEMO design configuration having a fusion power of 1998 MW and a tritium breeding ratio of 1.05 [15]. Being the blanket the region where tritium is produced and the fusion heat is removed through the primary coolant loops, it is characterized by high tritium concentration, high temperature and large metallic surface areas with reduced wall thickness. These features promote the tritium permeation from the blanket into the primary coolant. Once in the primary coolant, the tritium can reach the working areas and the external environment through permeation and leaks. For such reasons, the primary coolant loop has to be equipped with a system, named Coolant Purification System (CPS), dedicated to the tritium removal and control. Figure 1 illustrates the tritium migration path inside the DEMO reactor.

### 2.1. Triitum Mass Balance Inside the Helium Primary Coolant Loop

**Figure 2:**
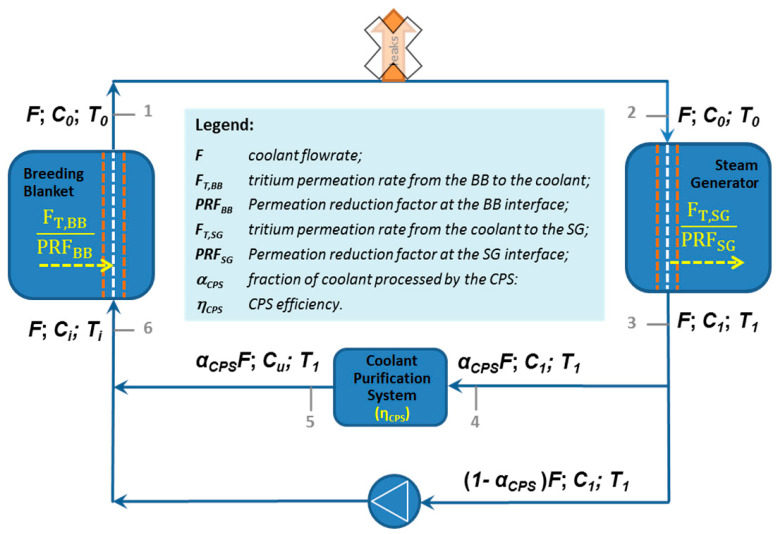
Simplified scheme of the coolant loop and CPS used for tritium mass balance.

A proper design of the CPS unit requires, as first step, the definition of the tritium mass balance inside the primary coolant loop. Figure 2 provides the scheme used to assess the tritium mass balance. As showed, the CPS is placed between the Steam Generator (SG) and the Breeding Blanket (BB) and it has to treat a certain by-pass of the helium primary coolant stream (α_CPS_) with a given efficiency (η_CPS_).

At steady state, the process can be divided into few steps, regulated by a set of mass balance equations. Starting immediately downstream of the BB (the point marked as 1 in Figure 2) and following (clockwise) the coolant flow all along the loop, it is possible to distinguish several segments:

(1-2) the coolant leaves the BB with a given flowrate F, tritium concentration C_0_ and temperature T_0_; if coolant leakages and heat losses are neglected, the coolant enters the SG with the same flowrate F, concentration C_0_ and temperature T_0_;

(2-3) while passing through the SG, the primary coolant releases heat to vaporize water in the secondary coolant loop, so that it leaves the SG at a temperature T_1_ < T_0_. Moreover, due to permeation across the SG heat exchangers, the tritium concentration in the primary coolant leaving the SG is also reduced to C_1_ < C_0_;

(3-4) a small fraction α_CPS_ of the total coolant flowrate F, with tritium concentration C_1_, is sent to the CPS, while all the remaining part flows through the bypass (3-6);

(4-5) the tritium concentration in the fraction of coolant processed by the CPS is reduced to a level C_u_ < C_1_, which depends on the purification efficiency (η_CPS_);

(5-6) the fraction of coolant flowing through the bypass (with a concentration C_1_) and that processed by the CPS, having a concentration C_u_ < C_1_, merge together upstream of the BB, resulting in a dilution of the tritium concentration at an intermediate value C_i_ (C_u_ < C_i_ < C_1_). The temperature T_i_ of the coolant at the inlet of the BB would be in principle lower than T_1_; however, if heat losses are neglected, we can assume T_i_ = T_1_;

(6-1) due to the heat release and tritium permeation from the BB, the coolant warms up and enriches in tritium concentration while passing through the BB, leaving this latter at the temperature T_0_ > T_i_ and with the tritium concentration C_0_ > C_i_.

The mass balance equations for the above described process are:

(3) $${\mathrm{FC}}_{1}={\mathrm{FC}}_{0}-\frac{F_{T,\mathrm{SG}}}{{\mathrm{PRF}}_{\mathrm{SG}}}$$

(4) $${\;\eta }_{\mathrm{CPS}}=\;\frac{C_{1}-C_{u}}{C_{1}}$$

(5) $${\mathrm{FC}}_{i}=\left(1-\alpha_{\mathrm{CPS}}\right){\mathrm{FC}}_{1}+\alpha_{\mathrm{CPS}}{\mathrm{FC}}_{u}$$

(6) $${\mathrm{FC}}_{0}={\mathrm{FC}}_{i}+\frac{F_{T,\mathrm{BB}}}{{\mathrm{PRF}}_{\mathrm{BB}}}$$

In some cases, tritium permeation can be reduced by covering the metallic surface with dedicated coatings or by enhancing the formation of oxide layers; the efficiency of these solutions in preventing the tritium permeation is indicated with the permeation reduction factor (PRF). In a first approximation, it is possible to neglect the tritium permeation rate from the primary coolant loop into the steam generator (i.e., F_T,SG_ = 0) and to assume the absence of antipermeation barriers in the blanket region (i.e., PRF_BB_ = 1). Under these hypotheses, by rearranging Equations (3)–(6), the amount of coolant to be processes inside the CPS is given by Equation (7).

(7) $${\;\alpha }_{\mathrm{CPS}}F=\frac{F_{T,\mathrm{BB}}}{\eta_{\mathrm{CPS}}C_{0}}$$

The above equation can be solved by considering as input that: (i) the tritium permeation rate from blanket into primary coolant (F_T,BB_) is approximately 0.7 g/day, as reported in Reference [16]; (ii) the tritium concentration inside the primary coolant loop has to be kept at 5 ppb (or lower) which corresponds to a pressure of 0.04 Pa, since the helium primary coolant is at 8 MPa; (iii) the efficiency of the CPS unit is estimated to be equal to 0.9. Using these inputs, the resulting coolant flow to be routed inside the CPS unit (α_CPS_) is about 3 kg/s.

### 2.2. A Coolant Purification System Based on NEGs

**Figure 3:**
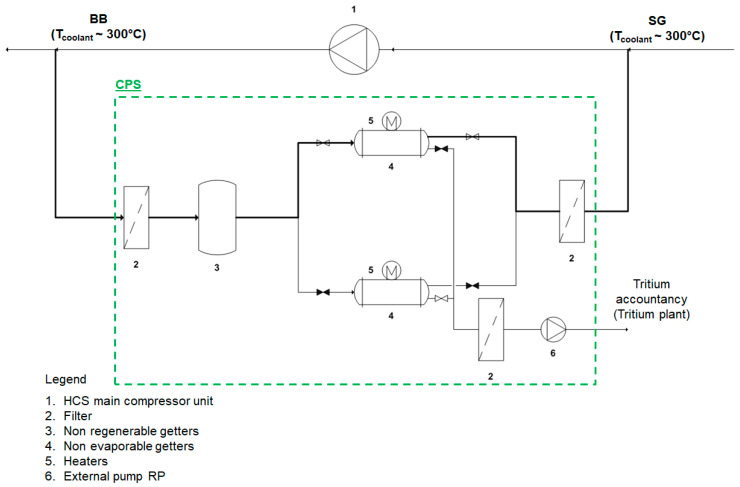
Scheme of the CPS pre-conceptual design based on novel Non-Evaporable Getter material.

Traditional processes for removing tritium from large helium flow rates foresee three separated steps: the HT oxidation into HTO using copper oxide beds, the subsequent HTO removal by means of molecular sieve beds and the final treatment of the HTO in dedicated systems [12,13]. However, these authors have recently proposed an option for tritium recovery from primary coolant loop, based on the use of NEGs technology [17,18]. In fact, NEGs material can directly adsorb the hydrogen species over their surface. This will prevent the need of the oxidation step and also the large cost associated to the processing of tritiated water. Figure 3 illustrates the CPS layout that uses NEGs for the recovery of the hydrogen species.

The operational mode of the proposed CPS layout foresees that the slipstream of the helium primary coolant loop, at first, encounters the inlet filter for dust and solid particulates removal and series of non-regenerable getter for impurities removal, then it goes into the NEG chamber for the removal of the hydrogen species, passes through another filter and then back to the main coolant loop. As better explained in the paragraph, the getter needs to be properly regenerated, for ensuring the continuous operation of the systems, at least two NEG chambers are required, one in operation and one in regeneration mode.

## 3. Feasibility Study on the Use of NEGs for Tritium Recovery from Helium on DEMO CPS Scale

To support a CPS layout proposal based on NEGs, the first action is to perform a feasibility study for identifying the most suitable metal alloy to be used and the amount required to fulfill the DEMO CPS requirements. The feasibility study has been carried out in agreement with the bulk getter theory, as fully described by Knize et al. in Reference [19] and has covered these different steps: (i) selection of suitable getter alloys, (ii) definition of the sorption flux regime, (iii) dimensioning of the NEGs at DEMO CPS scale and (iv) analysis of the regeneration parameters.

### 3.1. Selection of Suitable Getter Alloys

The process for identifying the most suitable NEG alloy composition for a given application, surely starts with the definition of the Sieverts’ constant and of the embrittlement limit. As shown in Equation (8), the Sieverts’ constant K(T) is a function of temperature T and of A and B parameters which depend on the specific NEG alloy:

(8) $$K\left(T\right)\;=\;10^{\left(A-B/T\right)}(\mathrm{torr}/(\mathrm{torr}\;l/g{)}^{2})$$

Regarding the embrittlement limit, it is worth to mention that NEG alloys are hydrides so they are subject to a progressive volume expansion during their operation and tend to flake and spall, producing small particulates. This embrittlement typically occurs when the amount of hydrogen isotopes sorbed and hence their concentration q, becomes high enough to modify the mechanical properties of the alloy. The concentration q_e_ at which this phenomenon occurs, clearly depends on the specific getter. The sorption capacity for hydrogen (i.e., the maximum quantity of this gas that can be sorbed by a given amount of a specific getter) is therefore limited by the embrittlement phenomenon. To prevent hydrogen concentration q from reaching the limit q_e_, NEG pumps need to be periodically regenerated, that is, heated and held at a suitably high temperature T_R_ (>T_S_) for a long enough time τ_R_ (both depending on the specific alloy) under dynamic vacuum (or under ultra-pure noble gas atmosphere) in order to release almost all the hydrogen sorbed. It is common practice to operate getters at a maximum hydrogen concentration about one half of their embrittlement limit.

Three different NEG alloys produced by the SAES group [20] have been considered. For each alloy, Table 1 illustrates the Sieverts’ parameters (A and B), the embrittlement limit (q_e_), the Sieverts’ constant (K(T)) and the hydrogen concentration at the getter surface (q_0_). Particularly K(T) and q_0_ have been assessed for two different temperatures (300 and 500 °C) corresponding to the temperature of the coolant before and after the steam generator. The values of q_0_ can be derived from Equation (9), by imposing a P_0_ equal to 3 × 10^−4^ torr (i.e., 5ppb of 8 MPa)

(9) $${\;q}_{0}\left(T\right)=\;{\left[P_{0}/K\left(T\right)\right]}^{1/2}=\;{\left[P_{0}/10^{\left(A-B/T\right)}\right]}^{1/2}$$

The Sieverts’ constant K(T), given by Equation (8), is a measure of the hydrogen solubility inside the getter material. Instead, Equation (9) shows that, for the same hydrogen partial pressure in the gas phase, the lowest is K(T), the highest is the hydrogen concentration that the getter can achieve. Among the getters listed in Table 1, ST101 exhibits the lowest value of K(T) and therefore the highest solubility, followed by ZAO and ST707, with ZAO that, however, has a significantly larger sorption capacity.

**Figure 4:**
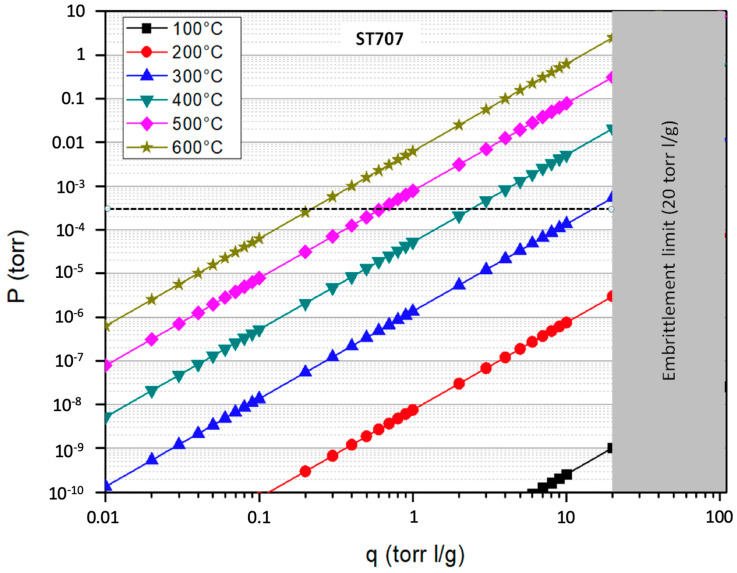
Sieverts’ plot of the ST707 alloy.

**Figure 5:**
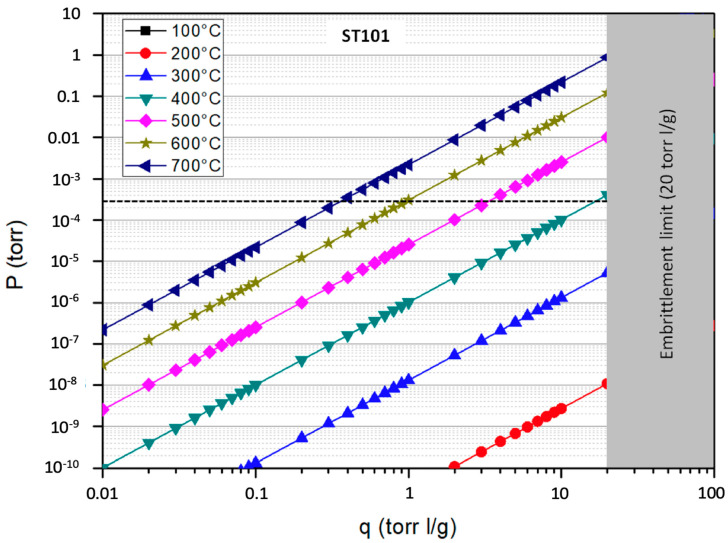
Sieverts’ plot of the ST101 alloy.

**Figure 6:**
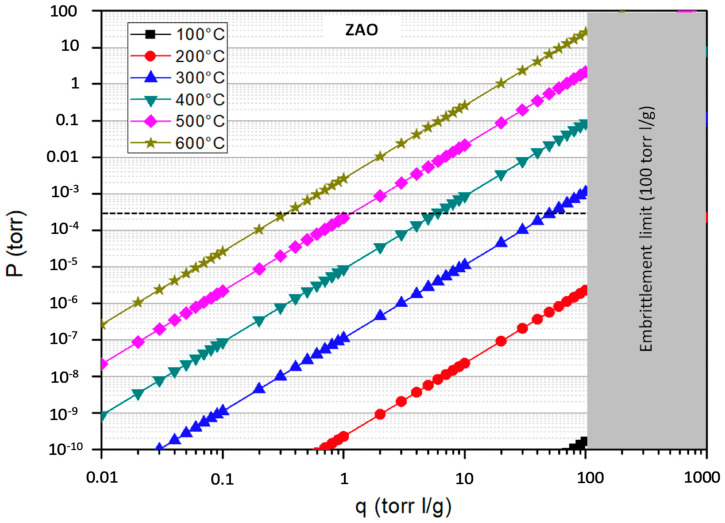
Sieverts’ plot of the ZAO alloy.

The Sieverts’ plots of the three alloys for different temperatures are reported in Figure 4, Figure 5 and Figure 6; the graphs also highlight the region above the embrittlement limit (in grey) and the value of the operative pressure (dashed line).

Common practice suggests operating the getters at an equilibrium concentration that not exceeds 50% of the embrittlement limit. Therefore, from the values reported in the Sieverts’ plots, it is possible to observe that ST707 and ZAO getters may be good candidates for a CPS located downstream of the SG where the operated temperature is about 300 °C. More precisely the sorption temperature of ST707 should be raised at a value of 320 °C to keep the tritium concentrations within this limit, that is, up to ~10 torr l/g, As for ZAO, to keep the equilibrium concentration within ~50 torr l/g, it would be sufficient to operate it at 302 °C but the difference is so small that can be neglected, so that ZAO getters can be operated at 300 °C, with no additional heating during sorption. Conversely the ST101 can be considered an option for CPS only if operated at a temperature of about 500 °C, corresponding to the coolant temperature before entering the SG.

In addition, it is important to notice that ZAO getters are particularly attractive due to their significantly higher sorption capacity (i.e., high q_e_ value) that can grant longer operation cycles and reduce the frequency of regenerations. On the other hand, ZAO alloy represents the latest developed intermetallic compound, therefore it is still poorly characterized.

### 3.2. Definition of the Sorption Flux Regime

The bulk getter theory provides different approximated solutions in the two limit situations corresponding to either a “surface limited” or a “diffusion limited” sorption flux. To decide which of these two regimes governs the process, it is necessary to evaluate the dimensionless parameter H, defined as the ratio of the surface limited flux, k_i_P_0_, to the diffusion limited flux, Dc_0_/L, see Equation (10).

(10) $$H=\;\frac{k_{i}P_{0}}{{\mathrm{Dc}}_{0}/L}$$

For H < 1, the sorption rate is limited by surface kinetics, whereas, for H > 1, it is governed by diffusion flux. The equilibrium concentration c_0_ (torr l/cm^3^) can be derived as follows:

(11) $$c_{0}\left(T\right)={\;\rho q}_{0}\left(T\right)\;(\mathrm{torr}\;l/{\mathrm{cm}}^{3})$$

where ρ is the getter density (g/cm^3^). The specific pumping speed k_i_ (i.e., the pumping speed per unit surface area) may depend on the particular getter and on its form; an appropriate order of magnitude is estimated to be ~0.1 L s^−1^ cm^−2^, for all the three different getters considered. The thickness L of getter layers deposited on metal substrate is typically ~100 µm. The diffusivity, D(T) in cm^2^/s, of hydrogen isotopes inside the bulk getter can be expressed in the Arrhenius form as:

(12) D(T)=exp(α−β/T)

The parameters α and β in Equation (12) depend on both the specific alloy and the gettered species, so, for the same getter, they are different for protium, deuterium and tritium. Data reported in literature for ST101 [21] and ST707 [22], are summarized in Table 2. Unfortunately, no data are available for tritium, so the same diffusivity of deuterium is assumed.

Regarding the ZAO alloy, since its characterization is still ongoing, α and β values are still under assessment. The information about its diffusion coefficient (D) at 300 °C has been provided to the authors directly by the SAES group.

The input values and the results of calculations aimed at estimating the dimensionless parameter H for the three alloys are summarized in Table 3.

In all cases H << 1 so, as expected, the sorption flux is limited by surface kinetics. Therefore, according with the bulk getter theory [19], the adsorption rate Γ_D_ (T,t) (in torr l s^−1^ cm^−2^) of a certain alloy can be approximated by Equation (13).

(13) $$\Gamma_{D}\left(T,t\right)={\;k}_{i}P_{0}\exp\left(-\frac{k_{i}P_{0}}{{\mathrm{Lc}}_{0}}t\right)$$

Since during the regeneration phase, the temperatures are higher than during sorption, the diffusivity is expected to further increase resulting in a reduction of the dimensionless parameter H. Therefore, the surface limited regime applies also in the regeneration phase. The sorption regime has been assessed using the diffusivity of deuterium. According to mass scaling law, the diffusivity of tritium is expected to be slightly lower, thus resulting in a marginally higher value of the dimensional parameter H, whose value for deuterium, however, is much lower than 1. We can therefore conclude that the sorption flux will be limited by surface kinetics even in the case of tritium.

### 3.3. Dimensioning of the NEGs at DEMO CPS Scale

Referring to the proposal of a coolant purification system for the DEMO reactor based on the use of NEGs (see Figure 2), the required mass (M) and area (A_G_) of a certain getter can be defined by fixing the follow boundary conditions: (i) at steady state, the amount of tritium ($\dot{Q}$) extracted per unit time from the getter surface should be equal to the amount of tritium that permeates from the blanket region into the primary coolant loop (see Equation (14)); (ii) too frequent regeneration cycles should be avoided, therefore the sorption time (τ_S_) should be at least greater than 1 day.

(14) $$\dot{Q}\left(T,t\right)={\;A}_{G}\Gamma_{D}\left(T,t\right)=F_{T,\mathrm{BB}}(\mathrm{torr}\;l/s)$$

For a given τ_S_, the amount of tritium adsorbed is provided by Equation (15).

(15) $$\Delta Q\left(\tau_{S}\right)=A_{G}{\overset{\tau_{S}}{\underset{0}{\int }}}_{D}\mathrm{dt}={\;A}_{G}k_{i}P_{0}\overset{\tau_{S}}{\underset{0}{\int }}\exp\left(-\frac{k_{i}P_{0}}{{\mathrm{Lc}}_{0}}t\right)\;\mathrm{dt}=A_{G}{\mathrm{Lc}}_{0}\left[1-\exp\left(-\frac{k_{i}P_{0}}{{\mathrm{Lc}}_{0}}{\;\tau }_{S}\right)\right]$$

Obviously, the mass of the getter (M) can be expressed as:

(16) $$M={\;\rho A}_{G}L$$

By combining Equations (14)–(16), it is possible to identify the mass and the area required for a given getter alloy (see Equations (17) and (18)).

(17) $$M=\;\frac{F_{T,\mathrm{BB}}\tau_{S}}{q_{0}\left[1-\exp\left(-\frac{k_{i}P_{0}}{{\mathrm{Lc}}_{0}}{\;\tau }_{S}\right)\right]}\;(g)$$

(18) $$A_{G}=\;\frac{M}{\rho L}=\;\frac{F_{T,\mathrm{BB}}\tau_{S}}{{\mathrm{Lc}}_{0}\left[1-\exp\left(-\frac{k_{i}P_{0}}{{\mathrm{Lc}}_{0}}{\;\tau }_{S}\right)\right]}\;({\mathrm{cm}}^{2})$$

**Figure 7:**
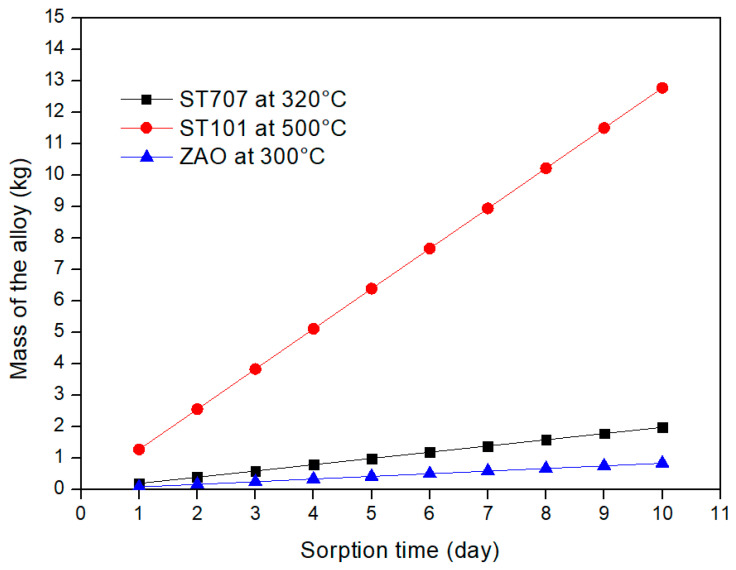
Mass of the different alloys required for DEMO CPS application vs. sorption time.

**Figure 8:**
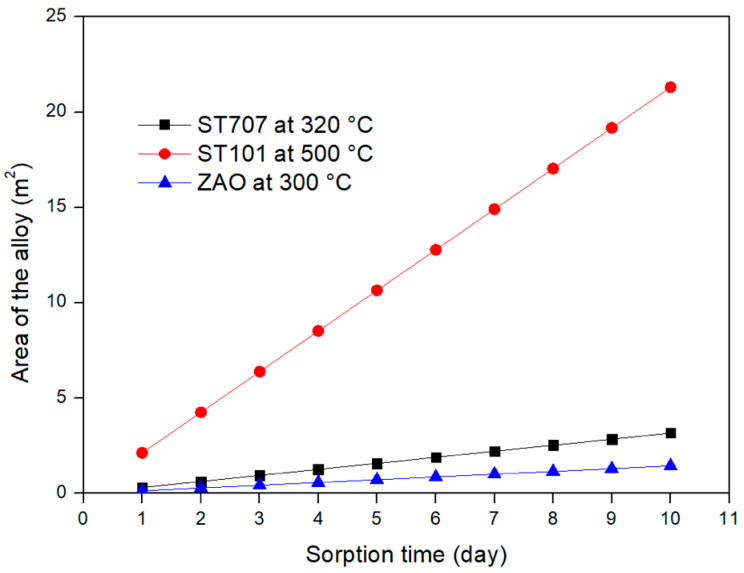
Area of the different alloys required for DEMO CPS application vs. sorption time.

For the three different alloys considered in this study, Figure 7 and Figure 8 provide the mass and the area required to fulfill the DEMO CPS requirements by considering a sorption time range between 1 and 10 days. The results clearly show that a DEMO CPS unit relying on the use of the ZAO alloy is the one that requires the lowest mass and area of the getter materials.

### 3.4. Analysis of NEGs Regeneration Parameters

To complete the feasibility study of NEGs for DEMO CPS application, the final aspect to be defined is related to the regeneration time (τ_reg_). Indeed, NEGs are storage pumps with a finite capacity, that can accumulate hydrogen into their bulk until they saturate. In order to release almost all the hydrogen sorbed and become ready for a new sorption cycle, getters need to be periodically regenerated at a temperature T_R_ higher than the sorption temperature T_S_. Therefore, to provide continuous operation, the CPS unit must be equipped with two (or more) getter beds, to ensure that, at any time, there is always one unit in operation, while the other(s) is(are) being regenerated. The overall time τ_reg_ required to regenerate a getter, is given by the sum:

(19) $$\tau_{\mathrm{reg}}={\;\tau }_{W}+\tau_{R}+\tau_{C},$$

where τ_W_ is the warm-up time, during which the temperature of the alloy is raised gradually (to avoid excessive thermal and mechanical stress) from T_S_ to T_R_, τ_R_ is the length of the regeneration plateau at constant temperature T_R_ and τ_C_ is the cool-down time, during which the temperature is reduced from T_R_ to T_S_. In practice, the regeneration starts soon after the end of a sorption cycle, when the getter has achieved equilibrium with tritium in the gas phase at a partial pressure P_0_, so that the initial concentration in the getter, at the beginning of the regeneration plateau, is ~q_0_, given by Equation (9) for T = T_S_. Similarly, at the end of the regeneration plateau, the residual tritium concentration in the getter must be equal to q_i_, where q_i_ << q_0_. The required time span of the regeneration plateau (i.e., the time needed to reduce the hydrogen concentration inside the getter from the initial value q_0_ to the final value q_i_), is given by:

(20) $$\tau_{R}=\;\frac{M}{S_{T}}\;\left[\frac{1}{q_{i}}-\frac{1}{q_{0}}\right]\frac{1}{K\left(T_{R}\right)}=\frac{M}{S_{T}}\;\left[\frac{1}{q_{i}}-\frac{1}{q_{0}}\right]10^{-\left(A-B/T_{R}\right)}$$

and depends on both q_i_ and q_0_, as well as on the getter mass M, on the regeneration temperature T_R_ and on the effective pumping speed S_T_ of the external pump.

In the following, different values of the regeneration temperature T_R_ have been considered and the corresponding values of the diffusivity D(T_R_), of the Sieverts’ constant K(T_R_) and of the warm-up time τ_W_, have been calculated. In particular for the warm-up time, τ_W_, has been estimated assuming a warm-up rate $\dot{T}$ of 2 °C/min. The results obtained for the three different alloys are reported in Table 4, Table 5 and Table 6.

Finally, the required length τ_R_ of the regeneration plateau have been calculated by considering a pumping speed S of the external backup pump of 1000 l/s and by imposing different values (from 0.1 down to 0.0001 torr l/g) for the final tritium concentration (qi) inside the three alloys at the end of the regeneration procedure. τ_0_ is defined as:

(21) $${\;\tau }_{0}={\;\tau }_{S}-\left(\tau_{W}+\tau_{R}\right).$$

As above, the calculations have considered different values of the regeneration temperature T_R_. The results obtained for the ST707, ST101 and the ZAO alloys are reported in Table 7, Table 8 and Table 9, respectively. Evidently, τ_0_ represents the time available, after the end of the regeneration plateau, for reducing the getter temperature from T_R_ to T_S_ and become ready, within the time τ_S_, for a new sorption cycle. Negative values of τ_0_ indicate that the sum of the warm-up time τ_W_ and of the regeneration plateau τ_R_ is longer than a sorption cycle τ_S_; this means that more than two getter beds have to be provided for ensuring the continuous operation of the CPS. Conversely, positive values of τ_0_ indicate that there is still some time available, after the end of the regeneration plateau, to allow for the cool-down phase (τ_C_). However, since this latter usually proceeds at a lower rate compared to warm-up, τ_C_ is expected to be longer (possibly of up to a few times) compared to τ_W_. For ensuring the continuous operation of the CPS unit composed of only two getter beds, τ_0_ must be longer than τ_C_, therefore the only cases of interest are those where τ_0_ is several times higher than τ_W_.

For the case in which the final tritium concentration, q_i_, inside the getter at the end of the regeneration procedure is set at 0.1 torr l/g, the results show that the lowest regeneration temperature considered for the three alloys (i.e., 650 °C for ST101 and 550 °C for ST 707 and ZAO) will allow to operate the CPS unit with only two getter beds. However it is important to notice that, for the case of ST707 and ST101, the results reported in Table 7 and Table 8 are obtained by considering a sorption time of 5 days (τ_S_ = 5 days) while for the ZAO alloy, due to its significant larger sorption capacity, it has been possible to consider a longer sorption time (τ_S_ = 10 days).

## 4. Discussion and Conclusions

This paper investigates an alternative application of the NEGs which are normally utilized in vacuum systems. The proposed application foresees the use of NEGs inside the coolant purification system of the EU DEMO reactor where a certain amount of tritium has to be removed from the primary coolant loop. Firstly the requirements of the coolant purification system have been defined. It has been assessed that about 3 kg/s of helium has to be continuously processed in order to maintain the tritium concentration inside the coolant loop at the value of 3 × 10^−4^ torr (5ppb). Then a feasibility study involving three different commercial getter alloys (ST707, ST101 and ZAO) from the SAES group has been conducted. According to the assessed Sieverts’ plot, the embrittlement limit and the operative pressure, the most appropriate sorption temperature for each alloys has been identified. Particularly the ST707 can be operated at 320 °C, the ST101 at 500 °C and the ZAO at 300 °C. Being the temperature of the helium coolant about 500 °C before entering the steam generator and about 300 °C when leaving, the ST101 can be considered an option for a CPS unit placed upstream the heat exchanger while the ST707 and ZAO are considered for an application downstream the heat exchanger. In such a way an additional heating of the getter during the sorption phase is not necessary. Then, after having verified that the sorption flux is surface-limited, the mass and the area of each alloy necessary to fulfill the CPS requirements have been assessed. By considering a sorption time of 5 days the required mass and area of the ST707 are about 1 kg and 1.6 m^2^, while for the ST101 are 6.4 kg and 10.6 m^2^. Since the ZAO alloy is characterized by a larger sorption capacity and an higher embrittlement limit, a longer sorption time has been considered; with a τ_S_ of 10 days the mass and the area necessary for the ZAO alloy are 0.8 kg and 1.5 m^2^, respectively. Finally the regeneration parameters have been evaluated by assuming different regeneration temperatures. This last evaluation has demonstrated that, under the investigated conditions, all the three alloys allow the operation of the CPS unit with two getter beds (one in operation and one in regeneration).

In conclusion, this study has demonstrated that the use of NEGs for the proposed application is feasible. Among the three alloys considered, the ZAO is the one that exhibits the best performances since it ensures longer sorption time, thus lower regeneration cycles. However the ZAO is a very recent material and requires additional experiments, particularly hydrogen diffusion parameters still need to be properly defined and adsorption/desorption parameters need to be assessed for the three hydrogen isotopes. In addition, future activities will be devoted to investigating the most appropriate NEG configuration. Traditional processes used for removing small amount of tritium from large helium flow rate foresee its oxidation into tritiated water that needs opportunely treated at a later stage. A great advantage of the proposed solution is that such oxidation stage is completely avoided.

## Figures and Tables

**Table 1:** Sieverts’ constants K(T) and surface equilibrium concentrations q_0_(T) at pressure P_0_ and at temperatures of 300 and 500 °C, for some common getter alloys.

P_0_ = 3 × 10^−4^ Torr	Sieverts’ Parameters		Embrittlement Limit q_e_ (Torr l/g)	Temperature T (°C)			
				300		500	
Getter Alloy	A	B		K(T) Torr/(Torr l/g)^2^	q_0_(T) (Torr l/g)	K(T) Torr/(Torr l/g)^2^	q_0_(T) (Torr l/g)
ST707 (Zr-V-Fe)	4.8	6116	20	1.34 × 10^−6^	14.98	7.73 × 10^−4^	0.62
ST101 (Zr-Al)	4.82	7280	20	1.30 × 10^−8^	151.74	2.52 × 10^−5^	3.45
ZAO (Zr-V-Ti-Al)	5.76	7290	~100	1.09× 10^−7^	52.46	2.13 × 10^−4^	1.19

**Table 2:** Diffusivity coefficients of H and D in ST101 and ST707.

	ST101		ST707	
	Protium	Deuterium	Protium	Deuterium
α	−2.9	−0.4	13.6	8.7
β	9900	12,100	18,700	14,600

**Table 3:** Coefficients used to establish the flux regime for the three different alloys (units are not in the SI but reflects the ones typically used in pumping applications).

		ST707 @ 320 °C	ST101 @ 500 °C	ZAO @ 300 °C
P_0_	[torr]	3.0 × 10^−4^		
k_i_	[l s^−1^ cm^−2^]	0.1		
k_i_P_0_	[torr l s^−1^ cm^−2^]	3.0 × 10^−5^		
L	[cm]	1.0 × 10^−2^		
K(T) = 10^(A-B/T)^	[torr/(torr l/g)^2^]	3.06 × 10^−6^	2.52 × 10^−5^	1.09 × 10^−7^
q_0_(T) = [P_0_/K(T)]^1/2^	[torr l/g]	9.89	3.45	52.46
ρ	(g/cm^3^)	6.26	6	5.8
c_0_ = ρq_0_	[torr l/cm^3^]	61.94	20.68	304.26
D (T) = exp(α-β/T)	[cm^2^/s]	1.22 × 10^−7^	1.07 × 10^−7^	5.16 × 10^−8^
Dc_0_/L	[torr l s^−1^ cm^−2^]	7.55 × 10^−4^	2.21 × 10^−4^	1.57 × 10^−3^
H = (k_i_P_0_)/(Dc_0_/L)	[dimensionless]	3.98 × 10^−2^	1.36 × 10^−1^	1.91 × 10^−2^
k_i_P_0_/Lc_0_	[s^−1^]	4.843 × 10^−5^	1.45 × 10^−4^	9.86 × 10^−6^
τ = Lc_0_/k_i_P_0_	[s]	2.065 × 10^4^	6.89 × 10^3^	1.01 × 10^5^

**Table 4:** Diffusivity, Sieverts’ constant and warm up τ_W_ of ST707 for different values of the regeneration temperature T_R_.

T_R_ (°C)		550	600	650	700
D(T_R_) (cm^2^/s)		1.186 × 10^−4^	3.275 × 10^−4^	8.104 × 10^−4^	1.827 × 10^−3^
K(T_R_) [torr/(torrl/g)^2^]		2.337 × 10^−3^	6.227 × 10^−3^	1.492 × 10^−2^	3.268 × 10^−2^
$\dot{T}$ (°C/min)		2	2	2	2
τ_W_	(hrs)	1.92	2.33	2.75	3.17
τ_W_	(min)	115.00	140.00	165.00	190.00

**Table 5:** Diffusivity, Sieverts’ constant and warm up τ_W_ of ST101 for different values of the regeneration temperature T_R_.

T_R_ (°C)		650	700	750	800
D(T_R_) (cm^2^/s)		1.358 × 10^−6^	2.664 × 10^−6^	4.892 × 10^−6^	8.489 × 10^−6^
K(T_R_) [torr/(torrl/g)^2^]		8.564 × 10^−4^	2.178 × 10^−3^	5.054 × 10^−3^	1.085 × 10^−2^
$\dot{T}$ (°C/min)		2	2	2	2
τ_W_	(hrs)	1.25	1.67	2.08	2.50
τ_W_	(min)	75.00	100.00	125.00	150.00

**Table 6:** Diffusivity *, Sieverts’ constant and warm up τ_W_ of ZAO for different values of the regeneration temperature T_R_.

T_R_ (°C)		550	600	650	700
D(T_R_) (cm^2^/s)^(#)^		1.186 × 10^-4^	3.275 × 10^-4^	8.104 × 10^-4^	1.827 × 10^-3^
K(T_R_) [torr/(torrl/g)^2^]		7.983 × 10^-4^	2.567 × 10^-3^	7.275 × 10^-3^	1.852 × 10^-4^
$\dot{T}$ (°C/min)		2	2	2	2
τ_W_	(hrs)	2.08	2.50	2.92	3.33
τ_W_	(min)	125	150	175	200

* Diffusivity is assumed to be the same as for ST707.

**Table 7:** Regeneration and cool-down times of ST707 for S = 1000 l/s and τ_S_ = 5 days.

**Length of the Sorption Cycle: τ*_S_* (days)**				**5**	**A_G_ (m^2^)**	1.583	**k_i_A_G_ (l/s)**	1.58 × 10^3^
Pumping speed of the backup pump: S (l/s)				1000				
T_R_ (°C)	550		600		650		700	
q_i_	Required length τ*_R_* of the regeneration plateau and time τ*_0_* available for cool down (hrs)							
(torr l/g)	τ*_R_*	τ_0_	τ*_R_*	τ_0_	τ*_R_*	τ_0_	τ*_R_*	τ_0_
0.1	1.90	116.18	0.71	116.95	0.30	116.95	0.14	116.70
0.01	19.20	98.88	7.21	110.46	3.01	114.24	1.37	115.46
0.001	192.19	−74.11	72.13	45.54	30.10	87.15	13.74	103.09
0.0001	1922.12	−1804.03	721.37	−603.70	301.06	−183.81	137.45	−20.62

**Table 8:** Regeneration and cool-down times of ST101 for S = 1000 l/s and τ_S_ = 5 days.

**Length of the Sorption Cycle: τ*_S_* (days)**				**5**	**A_G_ (m^2^)**	10.652	**k_i_A_G_ (l/s)**	1.065 × 10^4^
Pumping speed of the backup pump: S (l/s)				1000				
T_R_ (°C)	650		700		750		800	
*q_i_*	Required length τ*_R_* of the regeneration plateau and time τ*_0_* available for cool down (hrs)							
(*torr l*/*g*)	τ*_R_*	τ_0_	τ*_R_*	τ_0_	τ*_R_*	τ_0_	τ*_R_*	τ_0_
0.1	22.02	96.73	8.66	109.67	3.73	114.19	1.74	115.76
0.01	226.10	−107.35	88.92	29.42	38.31	79.61	17.85	99.65
0.001	2266.90	−2148.15	891.50	−773.17	384.09	−266.17	178.99	−61.49
0.0001	22,674.96	−22,556.21	8917.37	−8799.04	3841.91	−3723.99	1790.35	−1672.85

**Table 9:** Regeneration and cool-down times of ZAO for S = 1000 l/s and τ_S_ = 10 days.

**Length of the Sorption Cycle: τ*_S_* (days)**				**10**	**A_G_ (m^2^)**	1.449	**k_i_A_G_ (l/s)**	1.4485 × 10^3^
Pumping speed of the backup pump: S (l/s)				1000				
T_R_ (°C)	550		600		650		700	
q_i_	Required length τ*_R_* of the regeneration plateau and time τ*_0_* available for cool down (hrs)							
(torr l/g)	τ*_R_*	τ_0_	τ*_R_*	τ_0_	τ*_R_*	τ_0_	τ*_R_*	τ_0_
0.1	4.93	232.98	1.53	235.97	0.54	236.54	0.21	236.45
0.01	49.41	188.51	15.36	222.14	5.42	231.66	2.13	234.54
0.001	494.15	−256.23	153.65	83.85	54.22	182.86	21.30	215.37
0.0001	4941.58	−4703.67	1536.54	−1299.04	542.23	−305.15	212.97	23.70
